# Do Owners Have a Clever Hans Effect on Dogs? Results of a Pointing Study

**DOI:** 10.3389/fpsyg.2012.00558

**Published:** 2012-12-26

**Authors:** Teresa Schmidjell, Friederike Range, Ludwig Huber, Zsófia Virányi

**Affiliations:** ^1^Messerli Research Institute, University of Veterinary Medicine Vienna, Medical University of Vienna, University of ViennaVienna, Austria; ^2^Department of Cognitive Biology, University of ViennaVienna, Austria; ^3^Clever Dog Lab SocietyVienna, Austria

**Keywords:** Clever Hans effect, object-choice task, pointing gesture, dog

## Abstract

Dogs are exceptionally successful at interpreting human pointing gestures to locate food hidden in one of two containers. However, it has repeatedly been questioned whether dogs rely on the pointing gesture or their success is increased by subtle cues from their human handler. In two experiments we used a standard two-way object-choice task to focus on this potential Clever Hans effect. We investigated if and how owners’ knowledge and beliefs influenced their dogs’ performance. In two experiments, as is typical in such pointing tasks, the owners sat behind their dogs, in close auditory and tactile contact with them. In Experiment 1, we systematically manipulated the owners’ knowledge of whether or not their dog should follow the pointing gesture, but at the same time instructed the owners to refrain from influencing the choice of their dog. We found no influence of subtle cues from the owners, if indeed they existed: dogs in the different groups followed the pointing uniformly. Furthermore, in the absence of pointing dogs chose randomly, even though the owners had been informed about the location of the reward. In Experiment 2, owners were instructed to actively influence the choice of their dogs, and they, indeed, succeeded in sending their dogs to the container they believed to be baited. However, their influence was significantly weaker if the experimenter had previously pointed to the other location. Overall the pointing gesture seems to have a strong effect on the choice of dogs in an object-choice task. Pointing can lead the dogs to success without help from their owners as well as it can counteract clear directional instructions provided by the owners.

## Introduction

More than a century ago, a horse called “Hans” aroused interest in the field of animal behavioral research. He could answer mathematical questions by means of tapping his hoof. Testing the horse in various contexts, the psychologist Pfungst (1907) found that Hans could solve tasks by reacting to very subtle, subconscious cues of the questioner and the audience, such as their head jerks or body orientation, and thereby disproved the initial claim that Hans had a mathematical understanding.

Ever since Hans’ astonishing responsiveness to even the slightest human cues has made scientists cautious in interpreting the performance of animals in behavioral experiments involving human interaction. Most subjects of cognitive experiments grow up in close human contact (e.g., enculturated great apes, domesticated animals, socialized marine animals, and also human infants) and/or are repeatedly tested in tasks that involve interactions with humans. The experimenters usually know the correct solution expected from the subjects. Thus, instead of solving the given challenge on their own, the animals might respond to unintentional cues of the human participants. This so-called “Clever Hans effect” (Sebeok and Rosenthal, 1981) has led scientists to develop special methods in order to avoid such false positive results. For instance, special apparatuses have been designed to minimize contact between animal and experimenter (e.g., the Wisconsin test apparatus, Harlow and Bromer, 1938) or animals have been tested by projected images instead of interacting with a real human (Pongrácz et al., 2003). However, in some experiments direct contact with the subject may be a key element of the research question in focus, which makes complete exclusion of a human participant impossible. This is often the case when studying the interspecific socio-communicative abilities of dogs. In such studies, in order to exclude a potential “Clever Hans effect,” control experiments need to be included in which, for instance, the handler of the dogs is blindfolded while the dogs are provided with crucial information (e.g., Range et al., 2007; Hauser et al., 2011; Kaminski et al., 2011; Lit et al., 2011).

Presumably, the domestic dog is one of the animal species most susceptible to human-given cues. Dogs grow up in close contact with humans, and are often extensively trained to pay close attention to their human partners and to react to their behavioral cues (Serpell, 1996). Additionally, it has been suggested that during domestication they have been selected for increased attentiveness to humans (Hare and Tomasello, 2005), and dog-wolf comparisons have confirmed this hypothesis (Miklosi et al., 2003; Gácsi et al., 2009).

It has been demonstrated that dogs readily follow human-given cues to find hidden food (e.g., Miklósi et al., 1998; Hare and Tomasello, 1999; Soproni et al., 2001, 2002; Udell et al., 2008a), learn from a human experimenter how to solve manipulative tasks (e.g., Range et al., 2009), and take humans’ attentional cues into account (e.g., Call et al., 2003; Virányi et al., 2004). Usually, in these experiments the owner is holding the dog while the key elements of the experimental context are manipulated by an experimenter. Then the dog is released to respond. In experiments in which the response might be very simple, e.g., choosing one of two locations, the owner can possibly have a strong influence on the behavioral response of the dog.

A recent study by Hauser et al. (2011) suggested that the success of dogs in a pointing task could be influenced by cues provided by owners and experimenters. It did happen that the human participants accidentally deviated from the intended experimental procedure; e.g., the experimenters pointed for too long, the owners released the dog too early, or even tried to direct the dogs toward a specific container. Assuming that these mistakes did influence the choices of the dogs, Hauser et al. excluded many trials where a human coder could identify such mistakes. This led to a considerable loss of data, since 18.97% of all trials were excluded. They did not explicitly test, however, if these mistakes actually influenced the choices of the dogs’. This loss of data might therefore have been unnecessary if, contrary to their presumption, the mistakes had no effect on the dogs. On the other hand, it is possible that a dog is better at reading its owner’s behavioral cues than a human coder unfamiliar with the owner. Thus, despite of all the care taken, even the remaining data may still retain some human influence. Therefore, our aim was to explicitly test whether owners can actually influence the success of their dogs in such a pointing task.

Not only dogs have been tested in such pointing tasks but several other animal species as well, including domestic goats (*Capra hircus*; Kaminski et al., 2005), common bottlenose dolphins (*Tursiops truncatus*; Herman et al., 1999), South African fur seals (*Arctocephalus pusillus*; Scheumann and Call, 2004), African gray parrots (*Psittacus erithacus*; Giret et al., 2009), ravens (*Corvus corax*; Schloegl et al., 2007), gray wolves (*Canis lupus*; Udell et al., 2008b), coyotes (*Canis latrans*; Udell et al., 2012), dingoes (*Canis lupus dingo*; Smith and Litchfield, 2009), and red foxes (*Vulpes vulpes*; Hare et al., 2005). In these object-choice tasks the ability of the subjects to locate hidden food is tested after a human indicates with outstretched arm and finger which of two containers is baited with food. If the human-given pointing gesture is far away (>50 cm from the baited container) and is no longer present when the subject is finally released to make its choice, the gesture is called “momentary distal pointing” (for reviews, see Miklósi and Soproni, 2006; Reid, 2009). When having to find food based on this difficult gesture, the domestic dog outperforms even our closest non-human relative, the chimpanzee (*Pan troglodytes*; Hare et al., 2002; Bräuer et al., 2006). Since, in contrast to gray wolves (*Canis lupus*), dogs’ superior performance is already apparent by the age of 3 months, it has been suggested that evolutionary processes during domestication may have enhanced the socio-communicative abilities of dogs (Virányi et al., 2008; Gácsi et al., 2009). Making a step further, it has been argued that dogs and humans went through convergent evolution (Miklósi et al., 2004; Hare and Tomasello, 2005). Thus, ultimately the exceptional success of dogs in following human pointing has been used to make arguments about the evolution of human cognition (Hare and Tomasello, 2005; Hare et al., 2012). Due to the far-reaching theoretical impact of this simple test, it is of particular importance to question whether dogs outperform all other species in the pointing task because they became more cooperative and communicative during domestication, or because they are more responsive to subtle behavioral cues of their owners or other humans.

Consequently, here we set out to test whether and under what conditions owners can influence the success of their dogs to follow momentary distal pointing. As typical in such two-way object-choice task, the owners are seated behind their dog. We aimed to examine the potential effect of owner-given cues in the most likely case: between pet dogs and their owners who have been living together for at least 1 year. The dogs are likely to pay most attention to their owner (Horn et al., 2012) because of their emotional bond (Topál et al., 1998) as well as their life-long experiences with her (Jagoe and Serpell, 1996; Topál et al., 1997). Additionally, owners are likely to have the desire to support their dogs that they often regard as a family member or even a child substitute, and may also believe that the dogs require their help (Berryman et al., 1985; Albert and Bulcroft, 1988; Wan et al., 2009). Accordingly, similarly to others before us (Hauser et al., 2011; Kaminski et al., 2011; Lit et al., 2011), we assumed that owners might try to help their dogs either intentionally or unintentionally. For these reasons, it is likely that dogs rely on the behavior of their owners’ more than on that of an unfamiliar experimenter. Testing or controlling for potential experimenter-given cues is also crucial in experiments using human-animal interactions (Beran, 2012). In this study, however, we focused on potential owner influence because there is a high risk that we, experimenters, miss cues that the dogs are familiar with and can easily perceive via tactile, close auditory or olfactory contact with their owner during experiments.

## Experiment 1

The goal of the first experiment was to examine if owners affect their dogs’ choices after being instructed not to exert any influence upon their dogs. Hans responded to very subtle changes in his owner’s behavior, such as his rhythm of breathing or tension and relaxation. These changes would have been barely measurable, even when using video analysis. For this reason, we did not attempt to measure the owners’ behavior. Instead, we tested if the owners’ knowledge of the task influenced the performance of their dogs. To this aim, we tested four experimental groups, varying (a) the owners’ knowledge about the location of the food, (b) whether or not the experimenter pointed to a container, and (c) if the owner saw the pointing or not.

### Material and methods

#### Ethics statement

All procedures were performed in compliance with relevant laws and institutional guidelines. The owners and their dogs participated in this study on a voluntary basis. The daily testing procedure was short and entirely non-invasive. No special permission for use of animals (dogs) in such socio-cognitive studies is required in Austria.

#### Participants

Seventy-five dog-owner pairs participated in this experiment. Owners were women recruited through the Clever Dog Lab, Vienna. Six dogs were excluded due to motivational problems (not eating the food or not approaching the experimenter during pre-training) or due to deviations from the requested procedure by the owner (trying to influence the dog actively, e.g., by pointing toward a container, in spite of being instructed not to do so). The remaining sample of 69 dogs (32 males, 37 females; age: mean ± SD: 58 ± 30 months, range: 11–129 months) consisted of four different breed groups according to the FCI classification (sheepdogs and cattle dogs: *N* = 16; terriers: *N* = 10; retrievers: *N* = 15; companion and toy dogs: *N* = 9), as well as a group of mixed breed dogs (*N* = 19). All dogs were well-trained, having participated in obedience, agility, rescue, assistance, or dummy classes on a weekly basis. None of them had participated in a pointing experiment.

The owners had been asked not to feed their dogs 3–4 h before the experiment. Owners filled in a questionnaire to provide detailed information about their dog and their activities. Testing took place between December 2010 and June 2011.

#### Experimental set up and material

**Figure 1:**
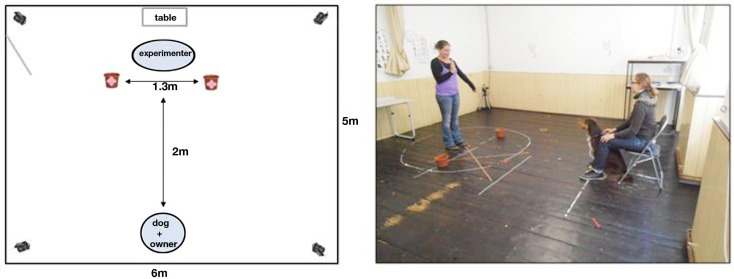
**Schematic drawing and photograph of the experimental set up, with the position of the owner (sitting behind the dog), the dog, and the experimenter, as well as the position of the four video cameras**. The pink signs on the containers in the drawing (left) indicate that both containers were always baited.

The experiment took place in the Clever Dog Lab, Vienna. The experimental equipment – consisting of a chair for the owner, a table for baiting the container – was arranged in a testing room (5 m × 6 m), as shown in Figure 1. The distance between the dog and the experimenter was 2 m. The positions of the experimenter and dog were delineated by tape markings on the floor.

As hiding locations, we used two identical brown plastic flower pots. Depending on the size of the dog, we used either smaller or bigger pots (larger dogs: *d* = 16 cm, *h* = 13 cm; smaller dogs: *d* = 13 cm, *h* = 10 cm). Note that, unlike in the standard pointing task, both containers were baited with small pieces of sausage or cheese in all trials. The reward was placed on the bottom of each pot in a way that the owners could not see them.

We recorded the behavior of the dog, the owner and the experimenter via four digital video cameras (2x Sony Exwave HAD, 2x Sony DCR-TRV 25) which were positioned in the four corners of the testing room. The cameras were connected to a video station (computerized recording system) outside of the testing room. It consisted of a Pinnacle Studio Moviebox creating an AVI output (720 × 576 High resolution video) that was recorded via the video station using the software, Virtual Dub.

### Experimental groups

Dogs were randomly assigned to one of four groups that were balanced for breed group, age, and sex. The groups varied in the owner’s knowledge about the location of the food, whether the owner could see the pointing or not and whether the experimenter did or did not point to a container (for an overview, see Table 1). The variation of these three factors leads to eight possible combinations. However, the four groups were chosen on the basis that if an influence would be present they would be most likely visible in these groups rather than in another combination that would not lead to meaningful results (e.g., Owner Blindfolded – no pointing – false knowledge).

**Table 1:** **Overview of four experimental groups**.

Group	O’s knowledge		E’s action (pointing)	O’s influence
Blindfolded *N* = 17	**- - - - - - - - - - - - - -**	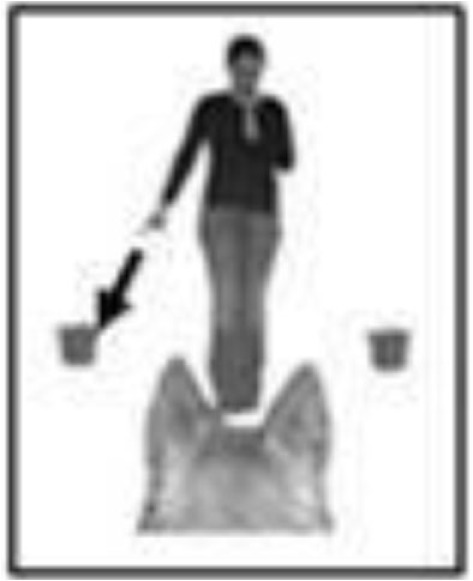	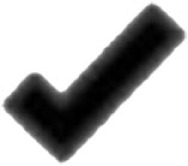	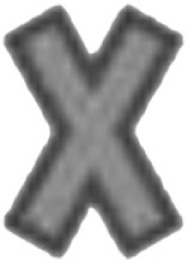
Enhancement *N* = 18	Dogs can follow pointing → Cueing to same side	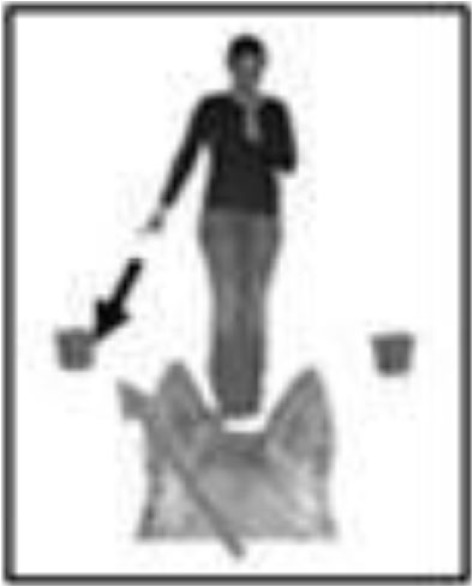	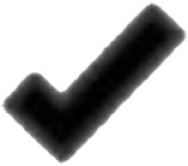	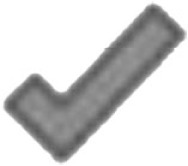
Decrease *N* = 17	Dogs can smell food in unpointed pot → Cueing to opposite side	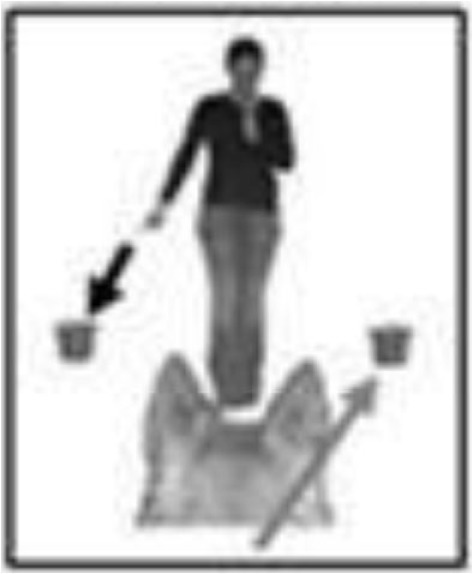	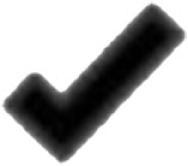	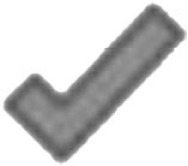
No Pointing *N* = 17	Knowledge about baited pot	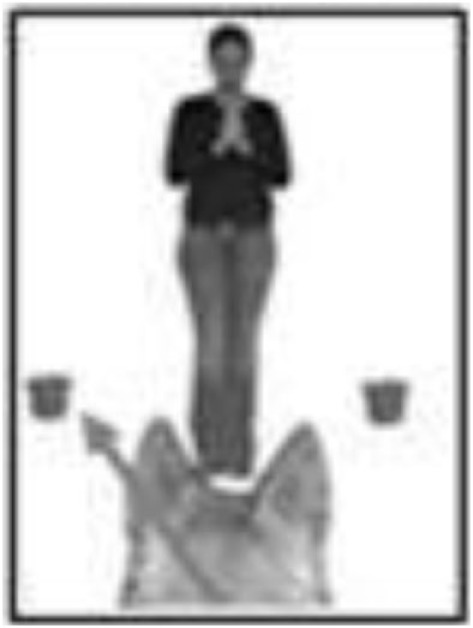	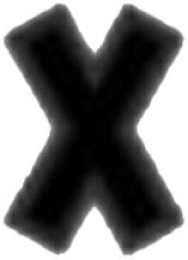	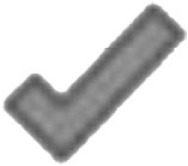

*O, owner; E, experiment*.

#### “Blindfolded” (*N* = 17)

In order to test whether dogs follow momentary distal pointing in the absence of potential owner-given cues, the owners were blindfolded and wore earphones during the test trials. That is, they did not see the pointing gesture and did not know where the food was. With this group we could also test if being rewarded over 20 trials irrespective of following the pointing or not would influence whether dogs follow the pointing gesture or not.

#### “Enhancement” (*N* = 18)

This group addressed the question whether or not the owners could increase the success of their dogs if they believed that their dogs could find food in the container the experimenter was pointing at. Accordingly, the owners were able to see the pointing and believed that there was food only in the pointed container. Since they had been told that their dog should follow the pointing, they might have subtly cued their dog to do so. Our question was if this would lead to a higher number of correct choices, as had been proposed earlier (Hauser et al., 2011).

#### “Decrease” (*N* = 17)

In this group we wanted to investigate if potential owner-given cues could decrease the number of correct choices dogs made in 20 momentary distal pointing trials. Owners were told that the objective was to test the dogs’ sense of smell and that their dog was expected to ignore the pointing gesture and to go to the other bowl. We predicted that the dogs’ performance should decrease in comparison to the blindfolded and enhanced group.

#### “No pointing” (*N* = 17)

Here we investigated whether, in the absence of a pointing gesture, owners could cue their dogs into choosing the container that the owners were told to be baited. We expected that if subtle cues were present and the dogs reacted to them, they would prefer the bowl the owner believed to be baited.

### General procedure

The general procedure (briefing, pre-training, testing, and debriefing) was the same for all dog-owner dyads. Importantly, across all groups and in every test trial, both containers contained an identical piece of reward [a small cube of soft sausage or cheese (1 cm^3^)] that the dogs could swallow quickly and silently, so that the owners could not see or hear if their dog was eating.

#### Briefing of the owners

In every group, the owners were told that only one container was baited (despite the fact that both containers were always baited) and that the aim of the study was to investigate if their dog succeeded in finding the hidden food. However, in the different experimental groups, we systematically varied the information the owner received (see below). Moreover, the experimenter also instructed the owner not to provide any helping cues to the dog. The participants received clear instructions where to seat their dog, to release the dog only when the experimenter was in a certain position (hands folded in front of her chest, head lowered), and not to point toward a specific side. The owners were allowed to praise their dog after each trial.

After the briefing, the experimenter led the owner and the dog into the experimental room and the owner released the dog to explore the room for 1–3 min.

##### Pre-training

The pre-training was conducted to familiarize the dog with the testing situation. The owner sat on the chair holding her dog by the collar. The dog sat in front of her facing the experimenter. The experimenter, standing at her position, simultaneously placed both containers on the floor, then stood up, and called the attention of the dog [by calling the dog’s name, and “*Schau!*” (engl.: “Look”)]. Once eye contact was established, she dropped a piece of food into one of the two containers in full view of the dog and the owner. She then folded her arms in front of her chest, put her hands together and lowered her head. As soon as she was in this position, the owner released the dog to approach a container. If the dog did not approach by itself, the owner was allowed to give a short command (e.g., “*Geh*” (engl.: “Go”). If the dog went to the correct container, the experimenter said “*Super, gut gemacht*” (engl.: “Super, well done”) in a praising voice. If the dog went to the wrong container the experimenter said *“Nein, leider, das war falsch”* (engl.: “No, that was wrong”). After the dog ate the food, the owner called it back to the starting position.

This procedure was repeated twice for each side. Before the dog could proceed to the testing phase, it was required to visit the correct location on four consecutive trials. A maximum of eight pre-training trials were performed.

##### Testing phase

The testing phase took place immediately after pre-training. Before each trial, the experimenter baited both containers with food, standing at the table with her back turned to the owner and the dog that could not see the baiting.

Each trial started with the experimenter placing the containers on the floor and standing up, facing the dog. In three of the four experimental groups (see Table 1), after calling the dog the experimenter pointed to one of the two containers (i.e., she stretched her ipsilateral arm with extended index finger toward the container for 2 s; the distance between her finger and the container was between 50 and 55 cm). In the “*No pointing*” group the experimenter told the owner which container would be baited. She then placed the containers on the floor, stood up and refrained from looking at the dog and from presenting a pointing gesture.

After this, the experimenter folded her arms in front of her chest and lowered her head. Then the owner released the dog to approach either of the containers. Importantly, in each trial of all experimental groups, both food containers were baited and the dog was allowed to eat the food no matter which container it visited. The experimenter started talking (either in a praising voice “super, well done” if the dog went to the container she had pointed to or “no, that was wrong” if the dog chose the other container) as soon as the dog had made a clear choice by placing their nose in a container. The owners could not hear or see that the dog was eating because the experimenter was talking, the dog faced toward the experimenter and the reward pieces were small and soft. In the “*No pointing*” group, the experimenter verbally reinforced the container that had previously been indicated to the owner as the correct one. After the dog had eaten the food from one of the containers, the experimenter picked both containers up and the owner called the dog back to the starting position.

Each dog received 20 trials with a break of 10 min after 10 trials. The side of the verbally reinforced container was pre-determined and semi-randomized, with no more than two consecutive trials occurring on the same side, and with an equal number of trials reinforcing the left and the right side.

##### Debriefing

After the testing phase the owner was asked to fill in a questionnaire (Table 2) in order to assess whether she realized that her dog was rewarded also for choosing the container that she believed to be empty. We wanted to know also if she remained to believe what the experiment told her about the aim of the test. The questionnaire recorded how many trials the owner believed her dog had succeeded in. In this way we could test if the owners assessed the performance of their dogs in accordance with their knowledge about the aim of the experiment. Owners could answer with the following possibilities: my dog succeeded in 0–5 trials, in 6–10 trials, in 11–15 trials, or in 16–20 trials. Afterward, the experimenter informed the owner about the true goal and methods of the study and in which group she had participated in.

**Table 2:** **Questionnaire owners filled in after the experiment**.

1. Was the explanation about the experiment sufficiently clear to you? Yes/No
2. Did you feel nervous during the experiment? Yes/No
3a. Was your dog nervous during the experiment? Yes/No
3b. If yes, did this influence his/her attention and therefore his/her performance negatively? Yes/No
4. Was it difficult for you to follow exactly the instructions of the experiment? Yes/No
5. Did the performance of your dog change in the 10 trials after the break compared to the first 10 trials? Yes/No
6. In how many of the 20 trials did your dog get the sausage? (possible answers: 0–5, 6–10, 11–15, 16–20; and if known exact number of successful trials)
7. Did you expect your dog’s performance? Why/Why not?
8. Could you have influenced the decision of your dog during the experiment? Yes/No. If yes, how?

### Detailed procedures of each experimental group

#### “Blindfolded”

Briefing: The experimenter told the owner that she would be participating in a pointing task, and, as is common in experiments on dog cognition, she would have to wear a blindfold and earphones to prevent giving any cues. The experimenter provided no explicit information how dogs usually perform in pointing tasks. After briefing and familiarizing the dog with the room, the owner received the blindfold and earphones. After each trial of the pre-training and testing the owners were permitted to remove the blindfold and earphones in order to call their dog and bring it back into position.

Testing phase: After the experimenter had presented the pointing gesture, crossed her arms and lowered her head, the owner received a signal through wireless earphones to release the dog. In this way we could avoid distracting the dog by other signals. The experimenter played the signal from a laptop, using a small remote control attached to her wrist.

#### “Enhancement”

Briefing: The owners were informed that they were participating in a standard pointing study. The experimenter explained how important it is for dogs to use this communicative signal in everyday life, and that it was well-known that dogs perform reliably in this task. That is, the owners were informed that their dog is expected to follow the pointing.

Testing phase: The experimenter presented a pointing gesture that the owners could see and believed to indicate the baited container.

#### “Decrease”

Briefing: The owners were informed that they were participating in a study in which we wanted to test the olfactory ability of dogs to find hidden food. During the explanation, the experimenter talked about the remarkable abilities of dogs in drug and explosive detection and in discriminating between twins with different body odor after eating different diets (Hepper, 1988). The experimenter further explained that she would always point to the empty container but we would still expected dogs follow their nose and go to the baited container. That is, the owners were told that their dog should go to the container the experimenter had not pointed to.

Testing phase: The experimenter presented a pointing gesture that the owners could see and believed to indicate the empty container.

#### “No pointing”

Briefing: Owners were informed that we were studying the decision making of dogs in a free choice situation presenting two containers, one of which was baited. Before each trial, the owner was verbally informed [*“rechts”* or *“links”* (engl.: “right” or “left”)] which of the containers was baited. The experimenter also informed the owners that both containers were rubbed with sausage and therefore dogs could not base their decision on their sense of smell.

Testing phase: The dog was allowed to make a choice without having seen a pointing gesture while the owner had a definite belief which container was baited.

#### Data analysis

The choices of the dogs were coded during the experiment and confirmed by subsequent video analysis. In the three groups with a pointing gesture, a correct choice was coded if the dog went to the container the experimenter had pointed to. In the “*No pointing*” group, a correct choice was coded if the dog went to the container the experimenter had named as baited before hand. Going to a non-indicated container was coded as an incorrect choice. If a dog made no choice (i.e., did not go to either container within 30 s) the trial was excluded from the analyses. Three dogs made a choice in only 19 trials out of 20 (two of them in the group *“Blindfolded*,” one in the group “*Decrease*”), 1 dog made a choice in 18 of 20 trials (in the group “*Blindfolded”*), and one dog made a choice in 17 of 20 trials (in the group “*Blindfolded”*). For each dog the total percentage of correct choices out of all choices made in a session was calculated.

To test whether the performance of the dogs changed over trials, we compared the number of correct choices in the first to the second session (10 trials before and after break) using a Wilcoxon matched-pair test. In addition we tested if the four experimental groups performed above chance level (Wilcoxon signed-rank test) and evaluated how many individuals in each group performed above chance level (binomial test).

To examine whether the owners’ belief of the aim of the study had an influence on the dogs’ performances in presence of pointing, we applied pair-wise comparisons (Mann–Whitney *U* test). We compared the *“Blindfolded”* group with the *“Enhancement”* and *“Decrease”* groups. A third pair-wise comparison between the *“Enhancement”* and *“No pointing”* groups was performed to examine the effect of the pointing gesture.

To examine owner beliefs in the *“Enhancement,”* *“Decrease,”* and *“No pointing”* groups, we compared the number of correct choices of the dogs to the number of trials the owners believed their dogs to be successful in (based on their answer given in the questionnaire). For this comparison, the actual choices of the dogs were classified in the same categories as those provided by the questionnaire (0–5, 6–10, 11–15, 16–20 “correct” choices) and these categories were compared with the owner’s assessment using a sign test. This analysis was not carried out in the “*Blindfolded”* group.

We applied non-parametric tests using SPSS 19. Test were two-tailed and considered significant if *p* < 0.05. A sequentially rejective Bonferroni correction was applied for multiple testing (Holm, 1979).

### Results and discussion

**Figure 2:**
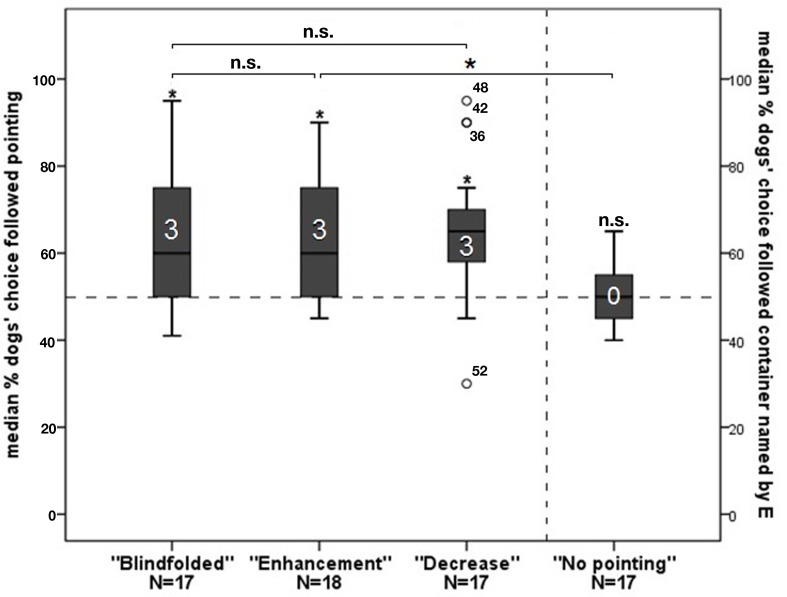
**The graph depicts box plots with the percentage of dogs’ choice**. Each box plot represents the spread of the sample and variability is indicated by the distance between the whiskers. Within the filled areas are 50% of all data, divided by the median into quartiles. Outliers are represented with circles. The first three bars represent the choice following the pointing gesture of the experimenter, the fourth bar represents choosing a container the experimenter previously named to the owner as baited. An asterisk directly above a bar indicates a significant difference in group performance from chance level; the number in the bar indicates the individuals in that group performing above chance.

Overall, dogs performed above chance level in all three groups with pointing (one-sample Wilcoxon signed-rank tests: “*Blindfolded”:* *N* = 17, *Z* = −2.924, *p* = 0.003; “*Enhancement”*: *N* = 18, *Z* = −3.140, *p* = 0.002; “*Decrease”:* *N* = 17, *Z* = −2.966, *p* = 0.003; all *p*-values Holm–Bonferroni corrected: *p* ≤ 0.05), whereas dogs in the group performed at chance level (one-sample Wilcoxon signed-rank test: *N* = 17, *Z* = −0.203, *p* = 0.839; Figure 2). At the individual level, in each of the groups with pointing three dogs performed above chance level (binomial tests: *p* < 0.05; Holm–Bonferroni corrected). Not a single dog in the *“No pointing”* group performed individually above chance (*p* > 0.05 before and after Holm–Bonferroni correction).

No significant differences between the first and second 10 trials of the ”*Enhancement”, “Decrease,” and ”No pointing”* groups were found (Wilcoxon matched-pair tests: “*Enhancement”*: *N* = 18; *Z* = 1.105, *p* = 0.269; “*Decrease”*: *N* = 17, *Z* = −1.655 *p* = 0.098; “*No pointing”*: *N* = 17, *Z* = 0.674, *p* = 0.500). However, in the “*Blindfolded”* group, the performance of the dogs decreased significantly from the first to the second session (Wilcoxon matched-pair tests*:* *N* = 17, *Z* = −2.776, *p* = 0.006; Holm–Bonferroni corrected: *p* ≤ 0.05).

*A priori* defined comparisons revealed that if a pointing gesture had been given, dogs performance neither increased (Mann–Whitney *U* test: *Blindfolded* vs. *Enhancement*: *N*_B_ = 17, *N*_E_ = 18; *U* = 151.5; *p* = 0.960) nor decreased (*Blindfolded* vs. *Decrease*: *N*_B_ = 17, *N*_D_ = 17, *U* = 157.0, *p* = 0.665) depending on the beliefs of the owners. This was also the case when comparing the performance of the dogs in the first 10 trials and the second session of these groups separately (*Blindfolded* vs. *Enhancement*: first 10 trials: *N* = 35, *U* = 116.500, *p* = 0.232; second 10 trials: *N* = 35, *U* = 193.500, *p* = 0.184; *Blindfolded* vs. *Decrease*: first 10 trials: *N* = 34, *U* = 154.500, *p* = 0.734; second 10 trials: *N* = 34, *U* = 157.500, *p* = 0.658).

Moreover, without pointing, even if their owners had been informed, dogs performed worse than when a pointing gesture had been presented (Mann–Whitney *U* test: *Enhancement* vs. *No Pointing*; *N*_E_ = 17, *N*_NP_ = 18, *U* = 236.0, *p* = 0.005; Holm–Bonferroni corrected ≤ 0.05; Figure 2).

Comparing the owners’ assessment of their dog’s success with the actual success of the dogs showed that the owners evaluated the actual performance of their dog differently in the different groups. Owners in the “*Enhancement”* group assessed the performance of their dog very similarly to its actual performance (sign test: *N* = 18, *p* = 0.250), and this was also the case in the *“No pointing”* group (sign test: *N* = 17, *p* = 0.625). In contrast, owners in the “*Decrease”* group assessed the performance of their dog significantly worse than it actually was (sign test: *N* = 17, *p* = 0.012). This shows that the owners all over the experiment believed that the correct response was to not follow the pointing.

Regardless of the owners’ belief, at a group level dogs followed the momentary distal pointing in all three groups where a pointing gesture was applied. Their performance was comparable to that of dogs tested previously in standard pointing tasks (e.g., Miklósi et al., 1998), which shows that having both containers baited did not influence the choices of the dogs.

Only in the group with the owner blindfolded did the performance of the dogs decrease as expected in all groups based on indiscriminate rewarding. Since dogs in the *“Decrease”* group continued to follow the pointing in the second 10 trials, it is unlikely that in the *“Blindfolded”* group the dogs learned not to follow the pointing. In this group the owners did not know when their dog performed well and when not, and thus, they encouraged their dogs less. Therefore, a more likely reason for the decreasing performance of dogs is that they lost motivation. It has been shown that encouragement plays an important role in cognitive experiments, and can influence the dogs’ performance (Topál et al., 1997; Horn et al., 2012). An alternative explanation might be that the dogs became increasingly uncomfortable because of unusual behavior of their owners. Since the owners were blindfolded and unable to hear what was going on, the dogs did not receive the usual reactions to their behavior. These results may confirm our assumption that dogs respond to subtle movements, tactile, or auditory cues of the owners that are unperceivable to us but can confuse dogs if they do not appear or appear in an unusual manner. However, the reason for the drop in the performance of the dogs remains speculative.

The owners’ assessment of the success of their dogs matched what our expectation based on their beliefs about the purpose of the study. Thus, it seems that they did maintain their belief across the entire experiment and did not notice when the dogs were rewarded with food. Despite this, the choices of the dogs were not influenced by the beliefs of the owners. The dogs followed the pointing gesture of the experimenter as long as it had been provided. On the other hand, when no pointing gesture had been presented but the owners had been informed about the location of the bait, the dogs performed at chance level, showing that they did not follow any possible cues of the owner. These results suggest that as long as the owners are instructed not to actively influence their dogs, no Clever Hans effect of the owners affected the performance of the dogs in this pointing task.

## Experiment 2

The results of the first experiment suggested that dogs followed the momentary distal pointing of the experimenter rather than potential helping cues of their owner. However, we cannot know if such inactive, potentially even subconscious cues of the owners were present at all. Furthermore, it is impossible to record the kind of owner influences dogs pay attention to. In order to investigate the extent to which the readiness of dogs to follow momentary distal pointing is susceptible to owner cueing, in a second experiment we examined how much the owners can actively affect the choice of dogs in the absence or presence of a pointing gesture.

### Methods

#### Participants

Thirty-six dog-owner pairs participated in this experiment. Owners were women recruited through the Clever Dog Lab, Vienna. Five dogs were excluded because of motivational problems (not eating the food or not approaching the experimenter during pre-training) or because the owner did not follow the experimenter’s instructions (e.g., sent the dog too early or called the dog back when not allowed). The remaining sample of 31 dogs (17 males, 14 females; age: mean ± SD: 56 ± 35 months, range 12–135 months) consisted of four different breed groups according to the FCI classification [sheepdogs and cattle dogs: *N* = 8; terriers: *N* = 4; retrievers: *N* = 8; companion and toy dogs: *N* = 3) as well as a group of mixed breed dogs (*N* = 8)]. All dogs were well-trained, having participated in obedience, agility, rescue, assistance, or dummy classes on a weekly basis. None of them had participated in a pointing experiment.

The owners had been asked not to feed their dogs 3–4 h before the experiment. Owners filled in a questionnaire to provide detailed information about their dog and their activities. Testing took place between December 2010 and June 2011.

#### Experimental set up and materials

The experimental set up was the same as in Experiment 1 (Figure 1).

### Experimental groups

Each dog was randomly assigned to one of two different groups that were balanced for breed group, age, and sex. The groups varied in the information the owners received about the experiment as well as the actions performed by the experimenter and the owner (for an overview, see Table 3).

**Table 3:** **Overview of the two experimental groups**.

Group	O’s knowledge		E’s action (pointing)	O’s influence
Owner active, *N* = 16	Dogs follow owner	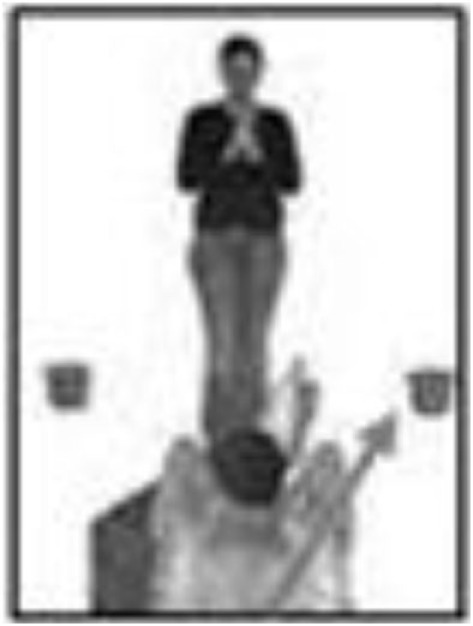	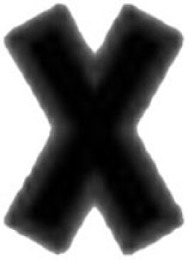	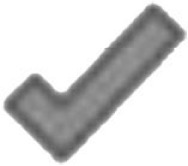
Pointing + owner active, *N* = 15	Against pointing → Opposite side	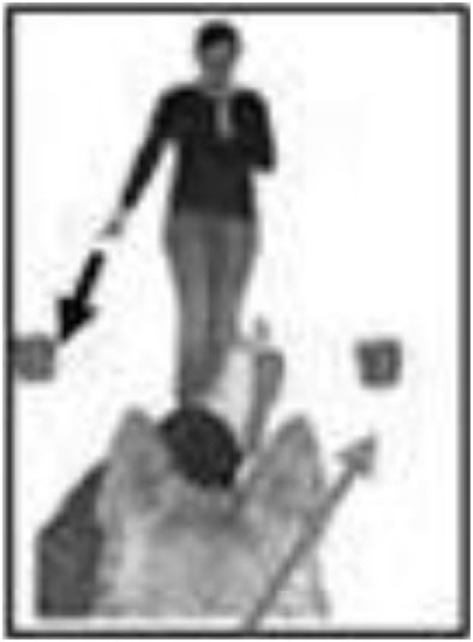	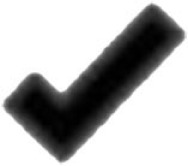	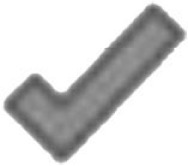

*O = owner, E = experiment*.

#### “Owner active” (*N* = 16)

Here we wanted to examine whether, in absence of experimenter pointing, owners could actively send their dog toward a container which they believed to be baited with food.

#### “Pointing + Owner active” (*N* = 15)

In this group we wanted to examine whether the owners could actively send their dog toward a container that they believed to be baited if previously the experimenter had pointed to the other container.

### General procedure

In both experimental groups the owners actively sent their dogs toward a container that they had been told to be baited after the experimenter either had or had not pointed to the other container. However, the general procedure (briefing, familiarization, pre-training, testing, and debriefing) was the same for all dog-owner dyads and identical to the procedure of Experiment 1. Importantly, in both groups and in every test trial both containers were baited as in Experiment 1.

#### Briefing of the owners

The owners were informed that only one container would be baited and that the aim of the study was to investigate if their dog would succeed in finding the hidden food. Each owner was instructed to actively send her dog toward the container which the experimenter had previously indicated to contain the food. The owner could use hand signals, pointing gestures, and vocal commands but was not allowed to stand up or move away from the chair. The owner was told not to influence or call the dog back if she had already released it and the dog was walking toward one of the two containers.

#### Pre-training

The pre-training was conducted as in Experiment 1.

#### Testing phase

After baiting both containers as in Experiment 1, each trial started with the experimenter placing the containers on the floor and standing up, facing the dog. In the “*Pointing* + *Owner active*” group (see Table 3), after calling the dog, the experimenter presented a momentary distal pointing gesture to one of the two containers, whereas in the “Owner active” group no pointing gesture was presented. In both groups, after the experimenter folded her arms in front of her chest and lowered her head the owner actively sent the dog and released it to approach one of the containers.

As in Experiment 1, during each trial both food containers were baited without the owner being informed about it and the dog was allowed to eat the food regardless of which container it visited. Eating the reward was concealed from the owner in the same way as in Experiment 1.

### Detailed procedures according to the experimental groups

#### “Owner active”

Briefing: Owners were informed that we wanted to see if dogs made their decision based on the owners’ active signals. The experimenter told them to try everything to make the dog go to the container where the food was hidden but not to call the dog back if it was already walking toward a container.

Testing phase: The experimenter indicated one of the containers (left or right) as baited before each trial, and then placed the containers on the floor and stood between them. Her hands were folded in front of her chest and her head lowered. As soon as the experimenter was in this position, the owner was allowed to send the dog to the pre-determined container. The experimenter praised the dog for choosing the previously indicated baited container. If the dog went to the non-indicated container the experimenter said “Nein, leider, das war falsch” (engl.: “No, sorry, that was wrong”), though the dog could nonetheless eat a piece of food reward there.

#### “Pointing + Owner active”

Briefing: Owners were informed that we wanted to test whether dogs choose a pointing signal given by the experimenter or active sending by the owner. The owners were told that the food was in the container which the experimenter did not point to. They were asked to try everything to send their dogs toward the baited (and non-pointed) container.

#### Testing phase

The experimenter presented a pointing that both the dog and the owner could see. As soon as the hand of the experimenter was back at her chest and her head lowered, the owner was allowed to try and send the dog to the other container. The experimenter praised the dog if it chose the pointed container. If the dog went to the owner’s target, the experimenter said “Nein, leider, das war falsch” (engl.: “No, sorry, that was wrong”) though the dog nonetheless could eat a piece of food reward there.

#### Data analysis

The choices of the dogs were coded during the experiment and confirmed by subsequent video analysis. A correct choice was coded if the dog followed the active cuing of the owner. Going to the other container was coded as an incorrect choice. In this experiment dogs never refused to make a choice. The percentage of correct choices was then calculated.

Since the experimenter verbally reinforced the dog it could also be the case that during the test the dog learned not to follow the instruction of the owner (even though the dog could eat food no matter which container it chose). Thus, we assessed whether or not a learning process occurred. To test whether the performance of the dogs changed over trials, we compared the number of correct choices in the first to the second session (10 trials before and after break) using a Wilcoxon matched-pair test. In addition we tested if the two experimental groups performed above chance level (Wilcoxon signed-rank test) and evaluated how many individuals in each group performed above chance level (binomial test). To reveal the effect of the pointing on the performance of dogs (i.e., following the owner), the two groups were compared (Mann–Whitney *U* test).

We applied non-parametric tests using SPSS 19. Test were two-tailed and considered significant if *p* < 0.05. A sequentially rejective Bonferroni correction was applied for multiple testing (Holm, 1979).

### Results and discussion

**Figure 3:**
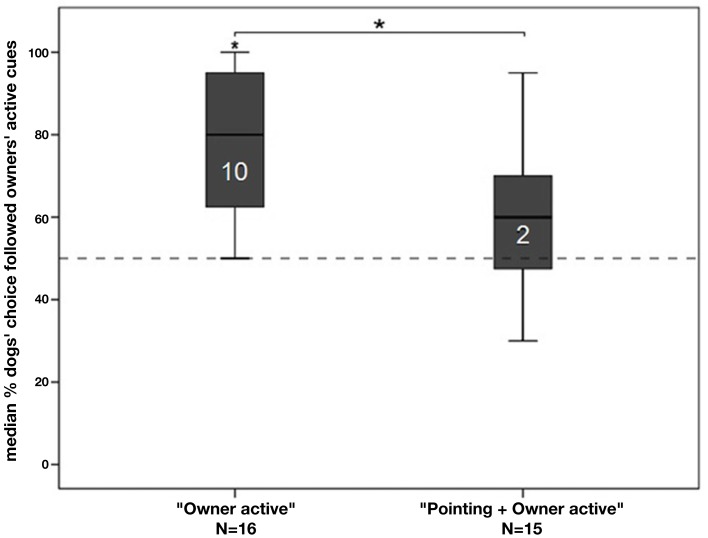
**The graph depicts box plots with the percentage of dogs’ choices to follow the owners’ target**. Each box plot represents the spread of the sample and variability is indicated by the distance between the whiskers, within the filled areas are 50% of all data, divided by the median into quartiles. An asterisk directly above a bar indicates a significant difference in group performance from chance level; the number in the bar indicates the individuals in that group performing above chance.

In none of the two groups did the % of correct choices in the first and second 10 trials (one-sample Wilcoxon matched-pair test: all *p*-values > 0.05), which suggests that the strategy of the dogs remained consistent across all trials. At a group level dogs in the “*Owner active”* group performed better than expected by chance (one-sample Wilcoxon signed-rank test: *N* = 16, *Z* = −3.417, *p* = 0.001; Holm–Bonferroni corrected: *p* ≤ 0.05) whereas the “*Pointing* + *Owner active”* group performed at chance level (*N* = 15, *Z* = −1.891, *p* = 0.059; Holm–Bonferroni corrected: *p* ≥ 0.05; Figure 3). Even though there was a trend indicating that dogs followed the instructions of their owner, the *p*-value remained non-significant after Bonferroni correction.

Analyzing the individual performance of dogs showed that 10 out of 16 dogs performed above chance in following their owner’s active cuing toward a container in the “*Owner active”* group (binomial test: *p* < 0.05; Holm–Bonferroni corrected). However, as soon as a contradictory pointing gesture had also been presented, only two dogs out of 15 followed their owner’s active cueing (binomial test: *p* < 0.05; Holm–Bonferroni corrected). No dog followed the pointing of the experimenter above chance level (binomial tests: *p* < 0.05; Holm–Bonferroni corrected).

There was a significant difference between the two groups: if there had been a pointing to the opposite container, owners were significantly less successful to send their dog to their target container (Mann–Whitney *U* test: *N* = 31, *U* = 48.500, *p* = 0.005; Holm–Bonferroni corrected: *p* ≤ 0.05).

## General Discussion

Overall, our results suggest that owners are only able to influence their dog’s choice in a two-way object-choice task if they are allowed to actively direct the movement of their dogs through the use of pointing gestures, physically pushing the dogs in the desired direction and/or commanding them. Owners that were instructed to refrain from such active manipulations had no measurable influence on the choice of their dogs, even though it is still plausible that they provided some subtle cues to influence their dogs to make a choice that they thought to be the correct or desirable.

In Experiment 1 we excluded only those dogs whose owners violated these instructions so strongly that the experimenter could easily identify their cuing behaviors during the experiment. These results suggest that in object-choice tasks using momentary distal pointing, the “Clever Hans effect” of owners does not have a significant influence on the number of correct choices of the dogs. In addition, we showed that the pointing gesture has a similarly strong effect to that of the active sending behavior of the owners, as shown in Experiment 2. The fact that dogs followed the owners’ active influence less if a pointing gesture had been presented is especially interesting, when considering that the pointing gesture was no more visible when the owners were allowed to send their dog toward the non-pointed container. However, since there was a time difference of only a few seconds between the pointing and the owners’ active influence one can say that pointing by an unfamiliar experimenter in the recent past and being sent by the owner right now had an influence of equivalent strength on most dogs.

These results question the assumption that the handlers of dogs can influence their behavior in two-way object-choice tasks. Based on the special bond owners form with their dog (Voith, 1985; Serpell, 1996, 2009; Zasloff, 1996;), the attachment that binds dogs to their owner (Topál et al., 1998; Gácsi et al., 2001; Prato-Previde et al., 2003; Palmer and Custance, 2008), and the plenty of experience dogs have with their owner and her/his behavior, the owner is likely to be the most effective person with regard to cueing their dog. Since we could not find a cuing effect of the owners, it is unlikely that a Clever Hans effect of the handlers of dogs plays a major role in other socio-communicative experiments using a two-way object-choice paradigm. This study, however, did not aim to investigate the possibility of a Clever Hans effect caused by the pointing experimenter. Since the experimenter (the first author) was aware of the goal of the study, she could have unintentionally influenced the choice of the dog. The pointing task primarily asks whether subjects can locate hidden food based on the cues of an experimenter that is in close proximity to the potential hiding places. Therefore, excluding the possibility that subjects follow cues other than the pointing gesture of the experimenter is often of lesser importance than excluding the possible explanation that the subject is directed by its handler. Nevertheless, testing the effects of potential unintentional experimenter-given cues by systematically manipulating the experimenter’s knowledge of the goal of the experiment and her expectations about the desirable behavior of the subjects is an important question and should be the aim of further studies using a double-blind design.

In our first experiment the owners were systematically manipulated in their beliefs about the goals of the study. Presumably their potential unintentional or intentional cues, if exist, are likely to reflected these. It might be argued that we should code the behavior of the owner to identify the specific behaviors that the dogs can use. However, we did not code this for two reasons. First, even if all visible behaviors of the owners could be analyzed [as in the study by Hauser et al. (2011)] it would still be possible that dogs are affected by cues that human observers cannot detect. The specific set up of two-way object-choice tasks facilitates tactile contact with the owner. Thus, not only visual cues can be involved in the process of information transfer potentially helping the dogs to go to the correct side. Second, it is likely that helping cues vary between dog-owner dyads (e.g., due to differences in training history), or that the type of owner personality or the specific dog-owner bond influences the cues used by the dyad. For example, several studies to date have shown that there are differences between the behavior of dogs according to the owners’ personality (Jagoe and Serpell, 1996; Kotrschal et al., 2009). Accordingly, we did not expect to find an overall consistent pattern of cueing. Nevertheless, based on the owners’ post-experimental reports it seems that we successfully manipulated their beliefs. Thus, it is reasonable to assume that cueing occurred in our experiment, even more, because we clearly informed the owners what behavior to expect from their dogs. This is usually not the case since experimenters typically refrain from informing owners about the goal of their studies in order to prevent cueing. Nevertheless, in simple cases, such as a pointing task, the owners can easily figure this information out themselves. An interesting question is to what extent the behavior of caretakers can be influenced by the verbal instructions or the style of an experimenter (Rosenthal, 1967; Silverman et al., 1972). A further interesting question can be whether cross-cultural differences exist in obeying experimental instructions (Blass, 2012). However, as argued earlier it is likely that the subconscious cues owners and dogs use are strongly influenced by the emotional aspects of their relationship. This was apparent in Topál et al. (1997) study that found that dogs with loose and strong attachment to their owners can solve manipulative problems with similar efficiency but attached dogs expect certain signals to do so. This is likely to be the case not only with obvious, but also subtle signals of permission and encouragement.

While keeping in mind that for these reasons our results cannot be easily generalized to other samples of dog-owner pairs, we form the tentative conclusion that momentary distal pointing (potentially accompanied by other subtle directional cues of the experimenter) has a strong influence on the choice of adult pet dogs in an object-choice task. This has been demonstrated by the results of both experiments as well as by finding that dogs in our task performed similarly well to other pointing studies, despite the fact that they were not differentially rewarded.

The possible mechanisms underlying dogs’ performance in pointing tasks have been discussed at length in former studies. One recent hypothesis assumes that dogs solve the pointing task due to local enhancement (e.g., being attracted to the protruding body parts; Lakatos et al., 2009, see also Udell et al., 2010). Although the method of momentary distal pointing ensures that local enhancement is reduced to a minimum, it still cannot be excluded that dogs follow the body parts that previously attracted their attention toward a certain place. Alternatively, dogs may succeed because they understand the communicative and referential character of the pointing gesture (for a review see Miklósi and Soproni, 2006). One set of results that supports this explanation is that dogs do not follow the pointing gesture if they do not expect to find food (Scheider et al., 2011). Very recently, Pettersson et al. (2011) set out to investigate the effect of varying communicative intention of the pointer – who was either cooperative (offering the subject food) or competitive (prohibiting the dog from taking the food). The comparison of these groups revealed no significant difference between them – indicating that the protruding pointing gesture may be most important and not the context in which it was given. However, in contrast to the cooperative context, dogs did not perform above chance in the competitive context; many dogs did not choose in the first trial or chose only after they were encouraged to do so. This however, is not sufficient evidence to argue that dogs perceive pointing as a communicative signal. Thirdly, it has been proposed that dogs perceive the pointing gesture as an imperative: a communicative signal instructing them to go to the pointed container (Kaminski, 2009; Topál et al., 2009). This hypothesis is supported by the finding that dogs follow the pointing gesture even after they saw that the other, non-indicated container was baited (Szetei et al., 2003; Erdöhegyi et al., 2007) and that dogs need a longer time to learn to go to the non-pointed than to the pointed container (Kundey et al., 2010).

At first glance, the results of the “*Pointing* + *Owner active*” group of our second experiment seem to contradict this later hypothesis. The sending actions of the owner (using positioning, pointing, verbal cues, and directing the dog manually) were clear imperatives directed at the dogs. Still, receiving these well-known instructions from the owner after having seen an experimenter-given pointing did not reliably determine the choice of the dogs. If dogs saw the pointing as an imperative then one could expect that the more recent imperative delivered by the owner could have superseded the experimenter’s antecedent pointing. This, however, was rarely the case. One may argue that pointing had a strong effect because the dogs expected food from the experimenter. This association might have already been formed during the pre-training (that was perceptually very similar to the testing phase) when the dogs might have learned to follow the indication of the experimenter to a certain location. The experimenter-food association does not mean, however, that the experimenter’s pointing gesture was perceived as referential communication. Possibly, it could have been seen as imperative which may lead to gaining a food reward if followed, and which could thus have the same strength in influencing the dogs’ behavior as the more recent instructions of the owner. To answer the questions of whether the pointing gesture counteracted the active influence of the owner or had the opposite effect, and of how the dogs would perform if the owners’ active influence was presented before the pointing gesture, further examination is necessary.

Although our study found no evidence of owners exerting a Clever Hans effect on their dogs in the presented object-choice task, this does not necessarily imply that owners would not cue the behavior of their dogs when presented with a different task or situation. Here the dogs were required only to make a simple choice: go left or right. Further studies, in which more sophisticated behaviors are analyzed, are required to create a comprehensive picture of the Clever Hans effect.

## Conflict of Interest Statement

The authors declare that the research was conducted in the absence of any commercial or financial relationships that could be construed as a potential conflict of interest.

## Appendix

**Table A1:** **number of choices (=following the pointing gesture/indicated container) for each individual**.

Dog’s name	Group	Followed pointing
Abby^b^	Blind folded	9
Akira	Blind folded	15
Archimedes	Blind folded	12
Bateia^a^	Blind folded	15
Cash	Blind folded	13
Chendra^c^	Blindfolded	7
Eddi	Blind folded	10
Keira	Blind folded	**19**
Lilyen	Blind folded	12
Luke	Blind folded	10
Mara	Blind folded	**17**
Nanook	Blind folded	11
Nemi	Blind folded	13
Olli	Blind folded	11
Poci	Blind folded	15
Quent	Blind folded	**16**
Timon	Blind folded	9
CD	Enhancement	12
Che	Enhancement	14
Chinua	Enhancement	11
Cool	Enhancement	9
Eshmoor	Enhancement	**17**
Heydi	Enhancement	9
Idefix	Enhancement	**18**
Ike	Enhancement	13
Jenny	Enhancement	**17**
Juki	Enhancement	13
Julie	Enhancement	10
Kelly	Enhancement	12
Lilly	Enhancement	12
Linette	Enhancement	15
Mala	Enhancement	9
Sam	Enhancement	15
Suki	Enhancement	12
Tina	Enhancement	10
Aika	Decrease	**18**
Archie	Decrease	12
Blacky	Decrease	13
Chester^a^	Decrease	11
Chilly	Decrease	12
Emy	Decrease	14
French	Decrease	**18**
Ginger	Decrease	14
Julie	Decrease	12
Keisha	Decrease	11
Luca	Decrease	9
Luis	Decrease	13
Max^a^	Decrease	**18**
Missy	Decrease	15
Momo	Decrease	13
Sokrates	Decrease	11
Zita	Decrease	6
Artos	No pointing	9
Arwen	No pointing	10
Axel	No pointing	10
Barolo	No pointing	11
Basti	No pointing	10
Blue	No pointing	13
Cookie	No pointing	13
Finlay	No pointing	9
Gundi	No pointing	9
Joey	No pointing	9
Kira	No pointing	8
Lele	No pointing	11
Micky	No pointing	12
Pebbles	No pointing	10
Samy	No pointing	9
Schnackerl	No pointing	9
Sky	No pointing	10

*^a^Indicates dogs that chose in 19 instead of 20 trials (*N* = 3)*.

*^b^Indicates the dog that chose in 18 instead of 20 trials (*N* = 1)*.

*^c^Indicates the dog that chose in 17 instead of 20 trials (*N* = 1)*.

*Numbers in bold indicate individuals performing better than expected by chance according to the binomial distribution. Note that due to Bonferroni – correction, this value was not the usual 15 out of 20, but instead 16 or more correct choices out of 20*.

**Table A2:** **Number of correct choices (=following the owners’ active cues) for each individual**.

Dog’s name	Group	Followed owner
Abby	Owner active	15
Buster	Owner active	**17**
Chili	Owner active	**20**
Elroy	Owner active	12
Flappi	Owner active	12
Flora	Owner active	**20**
George	Owner active	**16**
Indira	Owner active	10
Jessy	Owner active	**18**
Joey	Owner active	**19**
Knocky	Owner active	12
Lotti	Owner active	**16**
Mephisto	Owner active	**16**
Mika	Owner active	**19**
Shadow	Owner active	13
Tyrell	Owner active	**20**
Aika	Point ng + owner active	6
Amy	Pointing + owner active	14
Chester	Pointing + owner active	9
Diamond	Pointing + owner active	13
Flash	Pointing + owner active	12
Gala	Pointing + owner active	9
Chester	Pointing + owner active	7
Indigo	Pointing + owner active	**19**
Joy	Pointing + owner active	**16**
Nui	Pointing + owner active	14
Palmira	Pointing + owner active	14
Tango	Pointing + owner active	10
Timo	Pointing + owner active	15
Tiny	Pointing + owner active	10
Tosca	Pointing + owner active	10

*Numbers in bold denote individuals that performed better than expected by chance according to the binomial distribution. Note that due to Bonferroni – correction this value was not as usual 15 out of 20 but 16 or more correct choices out of 20*.

## References

[B1] Albert A. Bulcroft K. (1988). Pets, families, and the life course. J. Marriage Fam. 50, 543–552 10.2307/352019

[B2] Beran M. J. (2012). Did you ever hear the one about the horse that could count? Front. Psychol. 3:357 10.3389/fpsyg.2012.00357 23049522 PMC3448072

[B3] Berryman J. C. Howells K. Lloyd-Evans M. (1985). Pet owner attitudes to pets and people: a psychological study. Vet. Rec. 117, 659–661 4095881 10.1136/vr.117.25-26.659

[B4] Blass T. (2012). A cross-cultural comparison of studies of obedience using the Milgram Paradigm: a review. Soc. Personal. Psychol. Compass 6, 196–205 10.1111/j.1751-9004.2011.00417.x

[B5] Bräuer J. Kaminski J. Riedel J. Call J. Tomasello M. (2006). Making inferences about the location of hidden food: social dog, causal ape. J. Comp. Psychol. 120, 38–47 10.1037/0735-7036.120.1.38 16551163

[B6] Call J. Bräuer J. Kaminski J. Tomasello M. (2003). Domestic dogs (Canis familiaris) are sensitive to the attentional state of humans. J. Comp. Psychol. 117, 257–263 10.1037/0735-7036.117.3.257 14498801

[B7] Erdöhegyi Á. Topál J. Virányi Z. Miklósi Á. (2007). Dog-logic: inferential reasoning in a two-way choice task and its restricted use. Anim. Behav. 74, 725–737 10.1016/j.anbehav.2007.03.004

[B8] Gácsi M. Kara E. Belényi B. Topál J. Miklósi Á. (2009). The effect of development and individual differences in pointing comprehension of dogs. Anim. Cogn. 12, 471–479 10.1007/s10071-008-0208-6 19130102

[B9] Gácsi M. Topál J. Miklósi Á. Dóka A. Csányi V. (2001). Attachment behavior of adult dogs (*Canis familiaris*) living at rescue centers: forming new bonds. J. Comp. Psychol. 115, 423–431 10.1037/0735-7036.115.4.423 11824906

[B10] Giret N. Miklósi Á. Kreutzer M. Bovet D. (2009). Use of experimenter-given cues by African gray parrots (Psittacus erithacus). Anim. Cogn. 12, 1–10 10.1007/s10071-009-0277-1 18543008

[B11] Hare B. Brown M. Williamson C. Tomasello M. (2002). The domestication of social cognition in dogs. Science 298, 1634–1636 10.1126/science.1072702 12446914

[B12] Hare B. Tomasello M. (1999). Domestic dogs (Canis familiaris) use human and conspecific social cues to locate hidden food. J. Comp. Psychol. 113, 173–177 10.1037/0735-7036.113.2.173

[B13] Hare B. Tomasello M. (2005). Human-like social skills in dogs? Trends Cogn. Sci. (Regul. Ed.) 9, 439–444 10.1016/j.tics.2005.08.010 16061417

[B14] Hare B. Plyusnina I. Ignacio N. Schepina O. Stepika A. Wrangham R. (2005). Social cognitive evolution in captive foxes is a correlated by-product of experimental domestication. Curr. Biol. 15, 226–230 10.1016/j.cub.2005.01.040 15694305

[B15] Hare B. Wobber V. Wrangham R. (2012). The self-domestication hypothesis: evolution of bonobo psychology is due to selection against aggression. Anim. Behav. 83, 573–585 10.1016/j.anbehav.2011.12.007

[B16] Harlow H. F. Bromer J. A. (1938). A test-apparatus for monkeys. Psychol. Rec. 2, 434–436

[B17] Hauser M. D. Comins J. A. Pytka L. M. Cahill D. P. Velez-Calderon S. (2011). What experimental experience affects dogs’ comprehension of human communicative actions? Behav. Processes 86, 7–20 10.1016/j.beproc.2010.07.011 20696218

[B18] Hepper P. G. (1988). The discrimination of human odour by the dog. Perception 17, 549–554 10.1068/p170549 3244526

[B19] Herman L. M. Abichandani S. L. Elhajj A. N. Herman E. Y. K. Sanchez J. L. Pack A. A. (1999). Dolphins (Tursiops truncatus) comprehend the referential character of the human pointing gesture. J. Comp. Psychol. 113, 1–18 10.1037/0735-7036.113.4.347 10608559

[B20] Holm S. (1979). A simple sequentially rejective multiple test procedure. Scand. Stat. Theory Appl. 6, 65–70

[B21] Horn L. Virányi Z. Miklósi Á. Huber L. Range F. (2012). Domestic dogs (*Canis familiaris*) flexibly adjust their human-directed behavior to the actions of their human partners in a problem situation. Anim. Cogn. 15, 57–71 10.1007/s10071-011-0432-3 21739136

[B22] Jagoe A. Serpell J. (1996). Owner characteristics and interactions and the prevalence of canine behaviour problems. Appl. Anim. Behav. Sci. 47, 31–42 10.1016/0168-1591(95)01008-4

[B23] Kaminski J. (2009). “Dogs (*Canis familiaris*) are adapted to receive human communication,” in Neurobiology of “Umwelt”: How Living Beings Perceive the World, Research and Perspectives in Neurosciences, eds Berthoz A. Christen Y. (Berlin: Springer-Verlag), 103–107

[B24] Kaminski J. Nitzschner M. Wobber V. Tennie C. Bräuer J. Call J. (2011). Do dogs distinguish rational from irrational acts? Anim. Behav. 81, 195–203 10.1016/j.anbehav.2010.10.001

[B25] Kaminski J. Riedel J. Call J. Tomasello M. (2005). Domestic goats (Capra hircus) follow gaze direction and use social cues in an object choice task. Anim. Behav. 69, 11–18 10.1016/j.anbehav.2004.05.008

[B26] Kotrschal K. Schöberl I. Bauer B. Thibeaut A.-M. Wedl M. (2009). Dyadic relationships and operational performance of male and female owners and their male dogs. Behav. Processes 81, 383–391 10.1016/j.beproc.2009.04.001 19520238

[B27] Kundey S. M. A. De Los Reyes A. Arbuthnot J. Allen R. Coshun A. Molina S. (2010). Domesticated dogs’ (*Canis familiaris*) response to dishonest human points. Int. J. Comp. Psychol. 23, 201–215

[B28] Lakatos G. Soproni K. Dóka A. Miklósi Á. (2009). A comparative approach to dogs’ (*Canis familiaris*) and human infants’ comprehension of various forms of pointing gestures. Anim. Cogn. 12, 621–631 10.1007/s10071-009-0221-4 19343382

[B29] Lit L. Schweitzer J. B. Oberbauer A. M. (2011). Handler knowledges affect scent detection dog outcomes. Anim. Cogn. 14, 387–394 10.1007/s10071-010-0373-2 21225441 PMC3078300

[B30] Miklósi Á. Kubinyi E. Topál J. Gácsi M. Virányi Z. Csányi V. (2003). A simple reason for a big difference: wolves do not look back at humans but dogs do. Curr. Biol. 13, 763–766 10.1016/S0960-9822(03)00263-X 12725735

[B31] Miklósi Á. Polgárdi R. Topál J. Csányi V. (1998). Use of experimenter-given cues in dogs. Anim. Cogn. 3, 113–121 10.1007/s100710050016 24399275

[B32] Miklósi Á. Soproni K. (2006). A comparative analysis of animals’ understanding of the human pointing gesture. Anim. Cogn. 9, 81–93 10.1007/s10071-005-0008-1 16235075

[B33] Miklósi Á. Topál J. Csányi V. (2004). Comparative social cognition: what can dogs teach us? Anim. Behav. 67, 995–1004 10.1016/j.anbehav.2003.10.008

[B34] Palmer R. Custance D. (2008). A counterbalanced version of Ainsworth’s strange situation procedure reveals secure-base effects in dog-human relationships. Appl. Anim. Behav. Sci. 109, 306–319 10.1016/j.applanim.2007.04.002

[B35] Pettersson H. Kaminski J. Herrmann E. Tomasello M. (2011). Understanding of human communicative motives in domestic dogs. Appl. Anim. Behav. Sci. 133, 235–245 10.1016/j.applanim.2011.05.008

[B36] Prato-Previde E. Custance D. M. Spiezio C. Sabatini F. (2003). Is the dog-human relationship an attachment bond? An observational study using Ainsworth’s strange situation. Behaviour 140, 225–254 10.1163/156853903321671514

[B37] Pfungst O. (1907). Das Pferd des Herrn von Osten. Der Kluge Hans: Ein Beitrag zur Experimentellen Tierund Menschenpsychologie. Leipzig: Johan Ambrosius Barth [Engl.: Hans, C. (1911). *The Horse of Mr. von Osten: A Contribution to Experimental Animal and Human Psychology*, trans. C. L. Rahn (New York: Henry Holt)].

[B38] Pongrácz P. Miklósi Á. Dóka A. Csányi V. (2003). Successful application of video-projected human images for signalling to Dogs. Ethology 109, 809–821 10.1046/j.0179-1613.2003.00923.x

[B39] Range F. Heucke S. L. Gruber C. Konz A. Huber L. Virányi Z. (2009). The effect of ostensive cues on dogs’ performance in a manipulative social learning task. Appl. Anim. Behav. Sci. 120, 170–178 10.1016/j.applanim.2009.05.012

[B40] Range F. Virányi Z. Huber L. (2007). Selective imitation in domestic dogs. Curr. Biol. 17, 1–5 10.1016/j.cub.2007.04.026 17462893

[B41] Reid P. J. (2009). Adapting to the human world: dogs’ responsiveness to our social cues. Behav. Processes 80, 325–333 10.1016/j.beproc.2008.11.002 19056474

[B42] Rosenthal R. (1967). Covert communication in the psychological experiment. Psychol. Bull. 67, 356–367 10.1037/h0024529 6047176

[B43] Scheider L. Grassmann S. Kaminski J. Tomasello M. (2011). Domestic dogs use contextual information, and tone of voice when following a human pointing gesture. PLoS ONE 6:e21676 10.1371/journal.pone.0021676 21765904 PMC3135590

[B44] Scheumann M. Call J. (2004). The use of experimenter-given cues by South African fur seals (Arctocephalus pusillus). Anim. Cogn. 7, 224–230 10.1007/s10071-004-0216-0 15057598

[B45] Schloegl C. Kotrschal K. Bugnyar T. (2007). Modifying the object-choice task. Is the way you look important for ravens? Behav. Processes 77, 61–65 10.1016/j.beproc.2007.06.002 17644273

[B46] Sebeok T. A. Rosenthal R. (eds.) (1981). The Clever Hans phenomenon: communication with horses, whales, apes, and people. Ann. N. Y. Acad. Sci. 47, vii–viii, 1–309. 6942738

[B47] Serpell J. A. (2009). Having our dogs and eating them too: why animals are a social issue. J. Soc. Issues 65, 633–644 10.1111/j.1540-4560.2009.01617.x

[B48] Serpell J. A. (1996). Evidence for an association between pet behavior and owner attachment levels. Appl. Anim. Behav. Sci. 47, 49–60 10.1016/0168-1591(95)01010-6

[B49] Silverman I. Shulman A. D. Wiesenthal D. L. (1972). The experimenter as a source of variance in psychological research: modeling and sex effects. J. Pers. Soc. Psychol. 21, 219–227 10.1037/h0032331

[B50] Smith B. P. Litchfield C. A. (2009). Dingoes (*Canis dingo*) can use human social cues to locate hidden food. Anim. Cogn. 13, 367–376 10.1007/s10071-009-0287-z 19779743

[B51] Soproni K. Miklósi Á. Topál J. Csányi V. (2001). Comprehension of human communicative signs in pet dogs (*Canis familiaris*). J. Comp. Psychol. 115, 122–126 10.1037/0735-7036.115.2.122 11459158

[B52] Soproni K. Miklósi Á. Topál J. Csányi V. (2002). Dogs’ (*Canis familiaris*) responsiveness to human pointing gestures. J. Comp. Psychol. 116, 27–34 10.1037/0735-7036.116.1.27 11926681

[B53] Szetei V. Miklósi Á. Topál J. Csányi V. (2003). When dogs seem to lose their nose: an investigation on the use of visual and olfactory cues in communicative context between dog and owner. Appl. Anim. Behav. Sci. 83, 141–152 10.1016/S0168-1591(03)00114-X

[B54] Topál J. Miklósi A. Csányi V. (1997). Dog-human relationship affects problem solving behavior in the dog. Anthrozoös 10, 214–224

[B55] Topál J. Miklósi Á. Csányi V. Dóka A. (1998). Attachment behaviour in dogs (*Canis familiaris*): a new application of Ainsworth’s (1969) strange situation test. J. Comp. Psychol. 112, 219–229 10.1037/0735-7036.112.3.219 9770312

[B56] Topál J. Gergely G. Erdöhegyi Á. Csibra G. Miklósi Á. (2009). Differential sensitivity to human communication in dogs, wolves, and human infants. Science 325, 1269–1272 10.1126/science.1176960 19729660

[B57] Udell M. A. R. Dorey N. R. Wynne C. D. L. (2008a). Wolves outperform dogs in following human social cues. Anim. Behav. 76, 1767–1773 10.1016/j.anbehav.2008.07.028

[B58] Udell M. A. R. Giglio R. Wynne C. D. L. (2008b). Domestic dogs (*Canis familiaris*) use human gestures but not nonhuman non-human tokens to find hidden food. J. Comp. Psychol. 122, 84–93 10.1037/0735-7036.122.1.84 18298285

[B59] Udell M. A. R. Dorey N. R. Wynne C. D. L. (2010). The performance of stray dogs (*Canis familiaris*) living in a shelter on human-guided object-choice tasks. Anim. Behav. 79, 717–725 10.1016/j.anbehav.2009.11.033

[B60] Udell M. A. R. Spencer J. M. Dorey N. R. Wynne C. D. L. (2012). Human-socialized wolves follow diverse human gestures and they may not be alone. Int. J. Comp. Psychol. 25, 97–117

[B61] Virányi Z. Gácsi M. Kubinyi E. Topál J. Belenyi B. Ujfalussy D. (2008). Comprehension of human pointing gestures in young human-reared wolves (Canis lupus) and dogs (Canis familiaris). Anim. Cogn. 11, 373–387 10.1007/s10071-007-0127-y 18183437

[B62] Virányi Z. Topál J. Gácsi M. Miklósi Á. Csányi V. (2004). Dogs respond appropriately to cues of humans’ attentional focus. Behav. Process. 66, 161–172 10.1016/j.beproc.2004.01.012 15110918

[B63] Voith V. L. (1985). Attachment of people to companion animals. Vet. Clin. North Am. Small Anim. Pract. 15, 289–296 3872510 10.1016/s0195-5616(85)50301-0

[B64] Wan M. Kubinyi E. Miklósi Á. Champagne F. (2009). A cross-cultural comparison of reports by German Shepherd owners in Hungary and the United States of America. Appl. Anim. Behav. Sci. 121, 206–213 10.1016/j.applanim.2009.09.015

[B65] Zasloff R. L. (1996). Measuring attachment to companion animals: a dog is not a cat is not a bird. Appl. Anim. Behav. Sci. 47, 43–48 10.1016/0168-1591(95)01009-2

